# Periprosthetic Joint Infections

**DOI:** 10.1155/2013/542796

**Published:** 2013-08-19

**Authors:** Ana Lucia L. Lima, Priscila R. Oliveira, Vladimir C. Carvalho, Eduardo S. Saconi, Henrique B. Cabrita, Marcelo B. Rodrigues

**Affiliations:** Department of Orthopaedics and Traumatology, University of São Paulo, 05403-010 São Paulo, SP, Brazil

## Abstract

Implantation of joint prostheses is becoming increasingly common, especially for the hip and knee. Infection is considered to be the most devastating of prosthesis-related complications, leading to prolonged hospitalization, repeated surgical intervention, and even definitive loss of the implant. The main risk factors to periprosthetic joint infections (PJIs) are advanced age, malnutrition, obesity, diabetes mellitus, HIV infection at an advanced stage, presence of distant infectious foci, and antecedents of arthroscopy or infection in previous arthroplasty. Joint prostheses can become infected through three different routes: direct implantation, hematogenic infection, and reactivation of latent infection. Gram-positive bacteria predominate in cases of PJI, mainly *Staphylococcus aureus* and *Staphylococcus epidermidis*. PJIs present characteristic signs that can be divided into acute and chronic manifestations. The main imaging method used in diagnosing joint prosthesis infections is X-ray. Computed tomography (CT) scan may assist in distinguishing between septic and aseptic loosening. Three-phase bone scintigraphy using technetium has high sensitivity, but low specificity. Positron emission tomography using fluorodeoxyglucose (FDG-PET) presents very divergent results in the literature. Definitive diagnosis of infection should be made by isolating the microorganism through cultures on material obtained from joint fluid puncturing, surgical wound secretions, surgical debridement procedures, or sonication fluid. Success in treating PJI depends on extensive surgical debridement and adequate and effective antibiotic therapy. Treatment in two stages using a spacer is recommended for most chronic infections in arthroplasty cases. Treatment in a single procedure is appropriate in carefully selected cases.

## 1. Introduction

**Figure 1:**
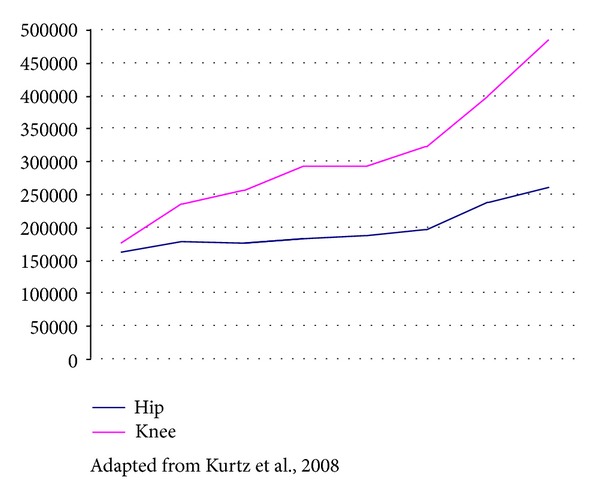
Evolution of the numbers of hip and knee prostheses implanted in the USA between 1990 and 2004.

**Figure 2:**
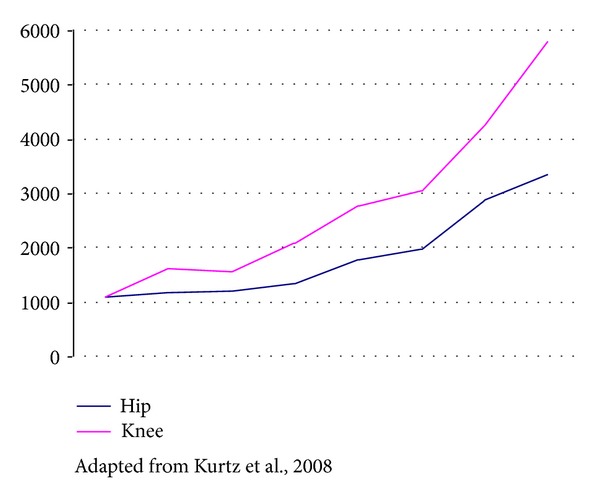
Evolution of the numbers of cases of prosthesis infection diagnosed in the USA between 1990 and 2004.

Implantation of joint prostheses is becoming increasingly common, especially for the hip and knee. It provides significant reduction in discomfort and immeasurable improvement in mobility for patients [1, 2]. It has been estimated that around 800,000 hip and knee prosthesis implantation procedures are performed only in the USA every year, counting both primary and revision surgery [3] (Figure 1). Although performed in smaller numbers, implantation of joint prostheses for the shoulder, elbow, wrist, and temporomandibular joint is also becoming more frequent [2]. From reviewing the worldwide literature, it can be seen that from 1 to 5% of these prostheses become infected, and it is important to bear in mind that as the number of operations for implanting these prostheses increases, so does too the number of cases that evolve with infection [3] (Figure 2). Although infection occurs less frequently than mechanical loosening does, infection is considered to be the most devastating of prosthesis-related complications, leading to prolonged hospitalization, repeated surgical intervention, and even definitive loss of the implant, with shortening or the affected limb and significant permanent deformity [1, 2]. 

## 2. Risk Factors and Physiopathogenesis

The main factors predisposing towards periprosthetic infection that have been cited in the literature are advanced age, malnutrition, obesity, diabetes mellitus, HIV infection at an advanced stage, presence of distant infectious foci and antecedents of arthroscopy or infection in previous arthroplasty [1, 2]. Patients with rheumatoid or psoriatic arthritis are also at greater risk of postoperative infection, which has been estimated to be three to eight times greater than among other patients. Prolonged duration of surgery (more than 150 minutes), blood transfusion, and performing bilateral arthroplasty during the same operation are other factors related to greater occurrence of infection. Any factor that delays surgical wound healing, such as ischemic necrosis, hematoma, cellulitis, or wound abscess, increases the risk of infection given that the deep tissues contiguous with the prosthesis do not have local defense barriers on the days subsequent to the operation [1, 2]. It is important to emphasize that the presence of the joint prosthesis leads to functional loss among the local granulocytes that accumulate around the implant, which become partially degranulated with diminished production of superoxide dismutase and loss of defense capacity against bacteria, particularly against *Staphylococcus aureus. *Thus, the presence of the implant decreases the size of the bacterial inoculum needed for infection to occur, by more than 100,000-fold [4].

Joint prostheses can become infected through three different routes: direct implantation, hematogenic infection, and reactivation of latent infection [2].

Microorganisms may penetrate the wound during the operation from both endogenous and exogenous sources. Examples of such sources include patient's cutaneous microbiota, microbiota of members of the surgical team, environment, and even contaminated implants.

Bacteremia from distant infectious foci may cause prosthesis contamination through a hematogenic route. The primary foci most frequently reported in the worldwide literature are the respiratory, cutaneous, urinary, dental, and gastrointestinal tracts [2, 4].

Gram-positive bacteria predominate in cases of joint prosthesis contamination, mainly *Staphylococcus aureus* and *Staphylococcus epidermidis*. Infections caused by Gram-negative bacilli and fungi such as *Candida sp. *are being reported more frequently around the world [4, 5]. 

## 3. Clinical Presentations and Diagnosis

 Periprosthetic joint infections present characteristic signs that can be divided into acute manifestations (severe pain, high fever, toxemia, heat, rubor, and surgical wound secretions) and chronic manifestations (progressive pain, formation of skin fistulae, and drainage of purulent secretions, without fever). The clinical presentation depends on the virulence of the etiological agent involved, the nature of the infected tissue, the infection acquisition route, and the duration of disease evolution. Several classifications have been proposed for defining the time at which contamination occurs and thus establishing the likely etiological agent involved and the best therapeutic strategy [1, 2, 4].

The classification system most widely used today is the one proposed by Fitzgerald Jr. et al., who divided infections related to arthroplasty as follows [6]:

- acute postoperative infections occurring within three months of the surgery. The etiological agents are generally of hospital origin, especially *S. aureus* and *S. epidermidis*;
- deep late infections that appear between three months and two years after the surgery. The etiological agents are considered to be of nosocomial origin, since the contamination probably occurred during the act of prosthesis implantation and generally consist of bacteria from the normal microbiota of the skin, such as *S. epidermidis* [7];
- late hematogenic infections that occur more than two years after the surgery. The etiological agents are of community origin and are determined by the apparent source of bacteria; dental infections are associated with bacteremia due to *S. viridans* and anaerobic bacteria, while cellulitis and skin abscesses are associated with *S. aureus* or streptococci. *Enterobacteriaceae *originate from the gastrointestinal and genitourinary tracts [8].

Despite nonspecific, C-reactive protein and erythrocyte sedimentation rates have shown sensitivity varying from 91% to 93%, respectively, and specificity varying from 86% to 83%, respectively, in patients with pain in knee arthroplasty and seem to be useful screening tools [9, 10].

**Figure 3:**
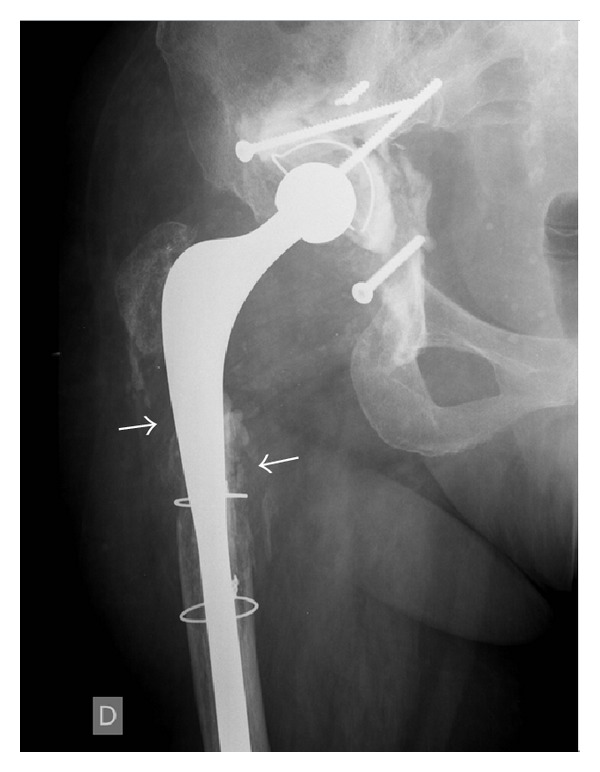
X-ray of total hip arthroplasty showing extensive lytic lesions around the femoral component (arrows), indicating infection.

The main imaging method used in diagnosing joint prosthesis infections is X-ray. The signs that suggest infection are a wideband of radiolucency at the cement-bone interface (in the case of cemented prostheses) or at the metal-bone interface (in uncemented prostheses), in association with bone destruction [11, 12] (Figure 3). However, it is generally not possible to distinguish between septic and aseptic osteolysis (relating to mechanical loosening/granulomatous disease) based on a single radiograph. Previous radiographs are needed for comparison [11, 13–15]. In cases of aseptic loosening, there is slow and progressive evolution, while in cases of infectious loosening, this loosening occurs rapidly, in a more aggressive manner and with greater bone destruction [16]. A plain radiograph should be performed in all patients with suspected prosthetic joint infection despite its low sensitivity and specificity because it can rule out conditions that could cause chronic pain [17, 18]. Nonetheless, there are cases of subclinical infection that also lead to loosening with slow evolution. The diagnosis of loosening can also be made by means of arthrography, such that in cases of either septic or aseptic loosening, the contrast injected into the joint ends up between the metal and the bone/cement. This method has the advantage that the joint fluid can be sampled for bacterioscopic evaluation and culturing [11, 19, 20].

**Figure 4:**
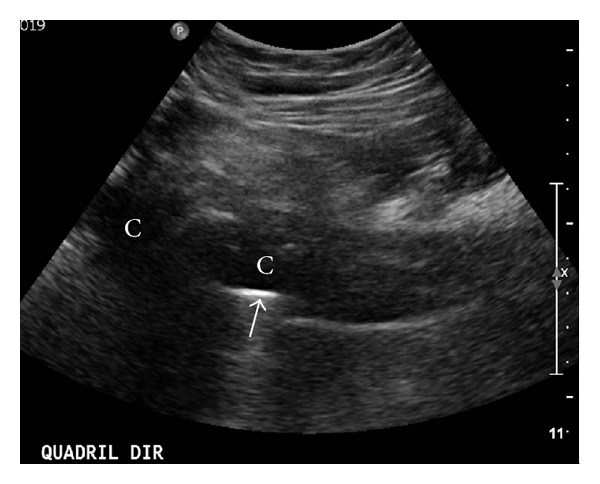
Ultrasound scan on a hip showing thick fluid collections (C) around the femoral component of the hip prosthesis (arrow).

 A computed tomography (CT) scan may assist in distinguishing between septic and aseptic loosening. The presence of a periosteal reaction or an accumulation of soft tissue adjacent to an area of osteolysis is highly suggestive of infection [21–23]. Ultrasonography may also be used to detect the presence of these soft-tissue fluid collections [23] (Figure 4). 

 The role of magnetic resonance imaging (MRI) is limited because of the artifacts generated by joint prostheses. Techniques for reducing the artifacts seen on MRI exist [24], but they are still generally not enough to enable adequate evaluation of the surrounds of the prosthesis [25–27].

 Some methods derived from nuclear medicine can also be used [28]. Three-phase bone scintigraphy using technetium has high sensitivity, but low specificity. Areas of high uptake may represent normal bone growth around the prosthesis, aseptic loosening or septic loosening. Bone scintigraphy has a high negative predictive value; that is, loosening (septic or aseptic) is practically ruled out if the scintigraphy result is normal. Use of gallium increases the diagnostic accuracy by 70%. Positron emission tomography using fluorodeoxyglucose (FDG-PET) presents very divergent results in the literature, with accuracies of 43 to 92% [29–31], and for this reason, it is not considered to be a reliable method for prosthesis evaluation. Scintigraphy using labeled leukocytes presents excellent results, with accuracy greater than 90%, and this is the scintigraphic method of choice for evaluating joint prosthesis infection. However, this method has low availability in clinical practice.

 Arthrocentesis should be considered in patients with suspected prosthetic joint infection when the diagnosis is not evident, there is clinical stability and surgery is not mandatory [17]. Patients with chronic painful prosthesis and elevated serum C-reactive protein or sedimentation rate should undergo arthrocentesis for diagnosis. Analysis of synovial fluid includes total cell count and differential leucocyte count and culture for aerobic and anaerobic organisms [17]. 

The definitive diagnosis of infection is made when the microorganism is isolated through cultures on material obtained from joint fluid puncturing, surgical wound secretions, or surgical debridement procedures [1, 2, 17], when there is a sinus tract in communication with the prosthesis, or when there is presence of purulence in the prosthesis [17]. During surgical debridement, five to six specimens should be sent to aerobic and anaerobic cultures [17, 32], and although mathematical models developed in a prospective study concluded that three or more cultures, with the same microorganism, should be positive for definite diagnosis of prosthetic joint infection [32], considering two or more positive cultures with the same organism or a single positive culture with a virulent microorganism has acceptable sensitivity and specificity [17]. Implants that are removed can also be subjected to sonication, and cultures on the solution in which this procedure is performed have been shown to be highly positive because of the capacity for isolating bacteria that separate from the biofilm during sonication on the extracted implant [2]; this procedure is more sensitive than cultures of periprosthetic tissue even when antibiotics are used within 14 days before surgery [33]. 

The use of molecular methods to diagnose prosthetic joint infection is the subject of several studies. The use of polymerase chain reaction (PCR) hybridization has been studied on implant subject to sonication for the diagnosis of prosthetic joint infection showing increase in final diagnosis, but false-positive results must be considered [34]. When 16S rRNA is used in intraoperative periprosthetic samples, the presence of the same microorganism in two of five samples results in sensitivity of 94% and specificity of 100%, and the presence of only one positive sample results in specificity of 96,3% and positive predictive value of 91,7% [35]. Real-time PCR has shown good correlation with infection severity [36]. Although promising, more studies must be carried out, and molecular technics should not substitute conventional methods [37]. The use of molecular diagnostics has applicability when conventional technics for microbiological diagnosis remain negative in the presence of fastidious microorganisms, infections due to *Mycobacterium spp.*, and infections acquired during the use of antibiotics [38].

## 4. Treatment

Success in treating periprosthetic joint infections depends on extensive surgical debridement and adequate and effective antibiotic therapy [2, 39, 40]. Infectious conditions that develop during the first year after the implantation procedure are considered to be surgical site infections and should be treated using broad spectrum antibiotics until the results from cultures on material collected in surgical debridement have been obtained. It is recommendable to start antimicrobial therapy at the time of induction of anesthesia, which avoids the risks to patients resulting from surgical manipulation of the infection focus that might exist without adequate coverage. Administered at this time, antibiotic therapy should not interfere with any positive findings on material collected during debridement. It is essential to have coverage against methicillin-resistant *S. aureus*, given the epidemiological importance of this agent in such infections [4, 39]. Total duration of antibiotic therapy ranges from six weeks to six months, and treatment should be adjusted whenever necessary, based on microbiological results [1, 2, 4]. 

It is important to consider that after two weeks of the contamination of the prosthesis, the bacteria adhering to the implant surface will be able to form sessile colonies, thus producing a layer of bacterial substances and necrotic tissue material called biofilm [41]. From this time onwards, it will no longer be possible to mechanically remove the bacteria from the implant, because of the protection coming from the polysaccharide surface layer of the biofilm, which is capable of resisting host defenses [42]. The environment surrounding the implant is devascularized and impedes direct action by antimicrobial agents [43].

Periprosthetic infections that appear within two to three weeks after the implantation procedure can initially be treated by means of extensive surgical cleaning in association with antibiotic therapy for six weeks [1, 2, 44, 45]. Infections that appear after this time, caused by biofilm formation and adherence of bacteria to the implanted material, should be treated by means of extensive surgical cleaning in association with removal of the joint prosthesis, which may be replaced in either a single procedure or in two stages. In the latter case, the total duration of antibiotic administration is six months [1, 2, 39, 40]. 

One-stage revision, which consists of removal of the infected prosthesis and immediate replacement with a definitive new prosthesis after debridement, was greatly used by European authors in the 1980s and 1990s [46]. Today, this procedure is contraindicated for infected patients with active fistulae, soft tissue in a poor condition, and bone losses that require bone grafts [47]. Besides, the site of prosthetic joint and the susceptibility of microorganisms to oral agents should be considered when one-stage revision is not formally contraindicated [17]. Indications for this procedure are generally conditional on implantation of cemented components. Thus, arthroplasty without cement should not be undertaken as a single procedure, although there are lines of research investigating implantation of prostheses without cement, and using bone grafts with added antibiotics [48–50].

Two-stage revision is the technique most used around the world [6, 51, 52]. Cure rates are greater than 90% after ten years of followup [53]. However, divergences exist regarding the ideal time interval between the initial surgery and implantation of the new prosthesis and regarding use of a spacer between the two stages.

**Figure 5:**
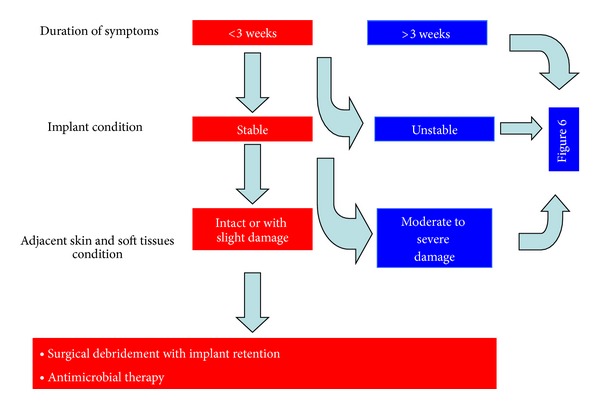
Management of acute periprosthetic joint infections.

**Figure 6:**
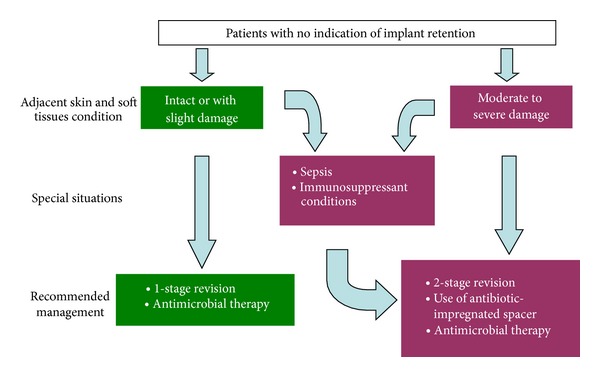
Management of periprosthetic joint infections with indication for implant removal.

Definitive removal of the implants with or without interposition of a muscle flap (Girdlestone procedure) or arthrodesis should be considered in severe cases of unstable patients [54, 55]. The following flow diagrams summarize the usual current recommendations for managing these infections (Figures 5 and 6).

Highest therapeutic success rates, which reach 93%, are related to the removal of the infected prosthesis associated with prolonged antimicrobial therapy, which should be chosen based on the etiological agent that was isolated from the material collected during the procedure to remove the prosthesis [39]. Polymethylmethacrylate (PMMA) impregnated with gentamicin or tobramycin can be used in cases of reimplantation of prostheses after infection. In cases of infection by methicillin-resistant *S. aureus*, PMMA can be impregnated with vancomycin, and use of high local doses of antibiotics has been shown to be effective in treating these types of infection [56, 57]. 

In summary, for treating chronic infections, implant replacement in two stages using a spacer is recommended for most cases. Treatment in a single procedure is appropriate in carefully selected cases. Randomized prospective studies are needed in order to achieve better definition of the boundary between indications for these two techniques, but two-stage treatment presents results that are more predictable and is a technique that can be used with greater security.
